# The Mouth Toilet

**Published:** 1915-07

**Authors:** Lyle C. Ling, Cecil R. Mull


### THE MOUTH TOILET.

READ BEFORE THE STUDENTS' DENTAL SOCIETY OF THE UNIVERSITY OF MICHIGAN, MAY 3, 1915.

BY LYLE C. LING AND CECIL R. MULL.

The subject of mouth toilet, as we all know, is one of the most discussed, or, we might say, it is the most discussed phase of dentistry today. Some, however, say that the reason is because it is the newest branch of the profession. This in a way, may be true, but the dental profession is rapidly beginning to see the value of the work that is being done along this line. From the present trend of medical science, it appears that an important branch of this work in the future will be that of aiding in the fight on tuberculosis. Dr. Robin Adair states that the vast majority of patients who have contracted tuberculosis, have unclean mouths. He also says that he believes the patient with the well-cared-for mouth is best able to resist this infection. The only successful treatment today known for tuberculosis, is that of fresh air, plenty of good food, pure water, and rest. The most important of these is proper feeding, and proper feeding depends upon proper mastication. Complete and proper mastication can not be accomplished unless the patient's mouth is in a healthy condition. Abscessed teeth, pus flowing from pyorrhea pockets, exposed pulps in teeth, and a considerable number of the teeth out of the jaw, or out of service, will not provide proper nutrition, even from the purest foods obtainable. The pure air of a pine forest, passing through a septic mouth, is no better than the air of a crowded tenement of a large city. Statistics show that full 70 per cent. of school children have enlarged tonsils and other glands. This means either a form of tuberculosis, or a predisposition towards that disease. A large per cent. of these can have none other than dental diseases as the source of these poisons, most of them from decayed teeth and open root canals. This has been demonstrated before the German Surgical Society by the process of innoculating the pulps of children's teeth. In the crusade against tuberculosis, there is not enough stress being laid upon oral sepsis as a causative factor for this disease, nor is importance enough attached to the value of oral sanitation as a cure of these patients. It seems as though this should be brought to the attention of the heads of the various institutions which treat tubercular conditions, also to the authorities who control charity institutions. It should be the duty of our profession to convince people of the great benefits that dentistry can accomplish for those under its care. The physician is always willing to show us how we may do better. A number of tubercular institutions have appointed dental clinicians to attend to the dental part of the work, but it seems that the majority of these men pay more attention to the filling operations while a very small per cent. of oral hygiene is done. Every patient who applies for dental attention should have his mouth cleansed with some antiseptic solution. The dentist should not examine any patient until this has been done. He should also apply to the teeth an iodine solution after they have been cleaned, and until all sepsis conditions are cured. Every patient should be instructed in the use of an adequate tooth toilet. If oral prophylaxis was advocated in this way the results would be convincing. Tubercular institutions would then feel more like appointing dental clinicians on their staffs for this purpose than they do at the present time. S. Adolph Knopf, of New York, says, "It must be said to the glory of American achievements that dental science, the art of preserving the teeth by scientific methods, had its birth in this country. While physicians go to Europe to complete their medical education, the European dentist comes to America to learn the best in his profession. Professor Fisher, of Yale, is authority for the statement "Seventy-two Americans die every hour from preventable diseases. Of these a large per cent. are due to dental origin, thus making it necessary for the dental profession to sit up and take notice. In this work it would be impossible for the dental profession or the medical profession to claim the whole field for the work, as it would be difficult for both combined to make a successful fight unless the people too work in co-operation and help fight "Preventable Diseases."

### BRUSHING THE TEETH.

Brushing the teeth is an essential part of the mouth toilet, and needs considerable consideration. It might be of interest to know that tooth brush handles at the present time are made of bone, purchased from the Chicago and other stock yards. The best grade handles being made from the thigh bones, while the cheaper ones are made from the shins and buttocks bone. The handles are turned by machines and then the back is grooved, the holes are drilled, and then wires are drawn through, which pull the bristles into place where they are cemented to place. The best bristles are obtained from Russia, India and Germany. As to the shape of the brush, there are any number of varieties, there also is a variation as to texture of the bristles. Nearly every dentist has some peculiar idea as to the shape and size of the brush. The teaching of the technic of brushing is, however, more nearly concerned with a proper mouth toilet. Few people today understand the proper method of brushing their teeth. This is due to the fault of the dentist in not imparting to them proper instruction. In some offices the dentists are especially interested in the method of using, and the kind of brush his patients should use, and have them for sale. This seems to be more satisfactory than sending them to a drug store where they probably will get the wrong kind. Other proper toilet articles, such as floss silk, dentrifice, and mouth washes might be sold by the dentist. It is a notorious fact that people do not buy brushes enough. They use them sometimes until they are almost worn to the handle. Such a brush is not only laden with germs of all kinds, but is little more than nothing with which to brush the teeth. Such a brush is always shedding its bristles, and may cause great irritation. Many dentists have what they call a ''direction card'' for handing to their patients with the sale of the brush. This would seem to be a good idea, for the dentist might instruct his patient orally and he would forget what was told him before he gets out of the office. Different ways of demonstrating are used. Drs. Edmund Kells and R. B. Adair have a full modeled artificial denture and they demonstrate to the patient, using this model. Others prefer to brush their own or the patient's teeth, having them hold a mirror so as to see the operation, and then have them do it after the method given. Training patients to brush their teeth properly seems to be one of the most thankless things the dentist has to do. The statement that a clean tooth will not decay means that the material on which germ life feeds has been removed, or materially decreased, and such cleanliness is the best prophylaxis we know. The tongue scraper, massage stick, and polish can be used by the patients. Tobacco stain can be kept from the teeth by means of the polishing stick and the floss silk tape, which will also prevent the formation of plaques. These devices are also of assistance to the mother or nurse in the care of children's teeth. The value of the tongue scraper was recognized by the Chinese many years ago, and a jeweled tongue spoon was part of their toilet requisites. The removal of disintegrated mucus from between the papillae of the tongue eliminates from the body a fine culture medium for all kinds of bacteria. This tongue scraper should be used shortly after arising each morning.

While on this subject it may be advisable to discuss briefly the bad breath question. It is very annoying to have some one near you at a social function, or while in a street car, or at any place that you might come in contact with them, and have an offensive breath blown into your face. The possessor of the bad breath often does not know of his affliction, and it seems as though he tries to get as close to your face as possible, while talking. Often these cases are of people who think they have taken some care with their mouth toilet. It is a very delicate subject to mention, but it would seem that the dentist could get around it better than any one else. It may not usually be discussed with the patient, owing to his sensitiveness on the subject; however, by taking a tactful method of speech we can suggest flossing the teeth to a better advantage or a frequent use of an antiseptic wash and the taking up by them of a system of prophylaxis.

Dr. Geo. M. Niles, a gastro-intestinal specialist, has written a valuable paper upon ''The Bad Breath and What It Portends.' It may be interesting to hear a few statements he makes in this article:

1. ''When the personal odor is offensive, it is a great misfortune, if preventable, it is an inexcusable disgrace.''

2. ''Every one of my readers can probably recall to mind one or more acquaintances who, except for an abominable breath, would be attractive, but because of the presence of this handicap, they are avoided, perhaps disliked.''

3. ''A busy dental surgeon of this city who has offices in the same building with a rectal specialist recently informed me, on comparing notes, they both decided that the dentist in his daily routine encountered more offensive and septic situations than did the latter in his rectal work.''

4. ''Efficient management by the physician or dental surgeon should afford relief and save embarrassment to the patient, and annoyance to his friends, and well may such emancipated sufferers rise up and call us blessed.''

While most of the cases of foul breath are due to mouth conditions of patients, they may arise in some degree from constipation, or intestinal intoxication; generally in uncomplicated cases in which the taking of some mild purgative medicine, such as a teaspoonful of epsom salts before breakfast, for a week or ten days, together with a large quantity of water, will help the condition. There are many different kinds of dentrifices and mouth washes, and it may be said that it isn't so much the brands as it is the way in which they are used. Some prominent dentists advocate lime water.

Oral prophylaxis has been advocated and practiced systematically since 1898. Dr. D. D. Smith, of Philadelphia, read a paper on Oral Prophylaxis at about that time and was the first dentist to advocate a systematic or professional form of treatment. At that time many criticised his work severely, believing that this kind of work should be done by the office assistant. Others said that the enamel would be polished away by the excessive treatments. From that time, however, this phase of dentistry has grown until now in every large city there are prophylaxis specialists. We find prominent dentists giving up their general practices to engage in this work. Prophylaxis treatment consists of enforced radical and systematic change of the environment of the teeth, and perfect sanitation for all the organs of the mouth. It means the most careful and complete removal of all extraneous calcic deposits, or semi-solid deposits, bacterial plaques, faulty secretions and excretions which gather on the surfaces of the teeth, between them, or at the gum margins. All fillings should be repolished, and any overhanging edges at the cervical borders should be made flush with the tooth surface, all badly fitting crowns and bridges should either be removed and replaced with better or be repaired and placed in a sanitary condition. After all the teeth have been properly scaled and cleaned, *every* surface should be polished with finely powdered pumice. But after the teeth and soft tissues of the oral cavity have been put in the most favorable condition, we have accomplished but very little if we stop here. We have only made a beginning. The patient must receive prophylaxis treatment every month, and he must be instructed by the dentist in the proper methods of brushing his teeth at home, and the use of mouth washes. After the first treatments, it does not require so much time or work. The treatment will consist mainly in cleaning the inaccessible places not reached by the tooth brush and the polishing appliances with pumice stone. The time between treatments varies much according to conditions which exist. Children should be treated at least once a month, and persons from 25 to 45, about once in three months. From 45 on, the treatment should be given once a month. Now, why is it necessary to have prophylaxis treatment today, when all these years up to the present time, cleaning the teeth once a year was thought to be all that was necessary? If you will go back a few generations, you will find conditions very different from those of the present day. In the first place, they lived more of an outdoor life, they took longer to eat their food. The cooks of those days were far different from ours. The cooks of today seem to have for their chief object, the preparation of food for absorption through the intestine, and to dispense, as it were, with the duties of the stomach. They try to prepare the food in as sticky a manner as possible. Seldom is it that we make a meal of such food that the teeth get to perform their real duties, such as tearing, rending and grinding. *The interproximal spaces in our mouth which were intended to be closed up, are now wide open to receive this starchy and other sticky food.* While we have this sticky mass adhering to the surfaces of the teeth, it constitutes the best pabulum for the growth of the numerous bacteria which are always in the mouth. And when once bacteria find a lodgment on some surface of the tooth, not cleaned by the brush, decay begins. Proper oral hygiene and prophylaxis will guard against decay but will not prevent it. A smooth polished surface of the tooth will not prevent bacteria collecting and lodging there. But this surface is more easily cleaned now. Now that a patient has been given systematic prophylactic treatment and carries out oral hygiene to the letter, what benefit is he going to get for it? He has derived many benefits.

1. The gums will resume their normal pink color and firmness.

2. Teeth that disfigure the face can be improved in appearance, for, if their surfaces be brought to a high state of polish, and the surrounding tissues healthy, one does not notice so much their ill shape.

3. Many defects in the teeth can be worked out, white spots removed.

4. Decay can be guarded against.

5. The vital structures within the tooth and those surrounding it, specially the peridental membrane, are maintained in normal condition.

6. The mouth is safe guarded against violent infections.

7. Pyorrhea is prevented.

8. Last but not least, the greatest result in prophylaxis is the training and maintenance by the patient at home of a perfect dental toilet.

Dr. Robin Adair kept a record, for the sake of comparison of his prophylaxis and his non-prophylaxis patients. He found that the non-prophylaxis patients had more caries and constantly recurring dental bills. Also he noticed the prophylaxis patients reported fewer doctor bills, and their illness, if any, has been light in character. Also they derived much pleasure from the knowledge that their mouths were in a healthy and beautifully polished state.

A man going into a dense forest engaged a guide; the guide had a belt, in which were a hatchet, a revolver, and three or four large knives. The guide clipped off a twig here and another there and after having repeated the operation a number of times, the gentleman said to the guide, "Why do you clip the branches?" He replied, "I am blazing the trail, so that when others come this way they will know where to go and how to follow, and this will be a guide for them." That is what we are doing, talking mouth hygiene, blazing the trail, teaching the laity what a healthy, clean mouth means. If they follow these instructions, it will aid them in conserving their teeth and health.

